# Correction: Atomic-scale engineering of Fe–Cu nanoparticles on amine-functionalized silica: CNT-driven synergy for ultra-efficient hydrogen evolution

**DOI:** 10.1039/d5ra90113h

**Published:** 2025-10-17

**Authors:** Nezar H. Khdary, Ekram H. El-Ads, Ahmed Galal, Ahmad O. Fallatah, Sami D. Alzahrani, Muteb F. Alotaibi, Mohammed J. Alotaibi

**Affiliations:** a King Abdulaziz City for Science and Technology Riyadh 11442 Kingdom of Saudi Arabia nhkdary@kacst.edu.sa; b Cairo University, Faculty of Science, Chemistry Department Giza 12613 Egypt

## Abstract

Correction for ‘Atomic-scale engineering of Fe–Cu nanoparticles on amine-functionalized silica: CNT-driven synergy for ultra-efficient hydrogen evolution’ by Nezar H. Khdary *et al.*, *RSC Adv.*, 2025, **15**, 33252–33263, https://doi.org/10.1039/D5RA03709C.

The authors regret that the name of one of the authors (Sami D. Alzahrani) was shown incorrectly in the original article. The corrected author list is as shown above.

The Royal Society of Chemistry apologises for these errors and any consequent inconvenience to authors and readers.

